# *In vitro* Study of *Bifidobacterium lactis* BL-99 With Fructooligosaccharide Synbiotics Effected on the Intestinal Microbiota

**DOI:** 10.3389/fnut.2022.890316

**Published:** 2022-04-28

**Authors:** Qi Zhang, Wen Zhao, Yuyang Zhao, Sufang Duan, Wei-Hsien Liu, Chao Zhang, Siyuan Sun, Tingting Wang, Xin Wang, Wei-Lian Hung, Ran Wang

**Affiliations:** ^1^Department of Nutrition and Health, Key Laboratory of Functional Dairy, Co-constructed by Ministry of Education and Beijing Government, China Agricultural University, Beijing, China; ^2^Beijing Laboratory of Food Quality and Safety, College of Food Science and Nutritional Engineering, China Agricultural University, Beijing, China; ^3^Inner Mongolia Dairy Technology Research Institute Co., Ltd., Hohhot, China; ^4^Yili Innovation Center, Inner Mongolia Yili Industrial Group Co., Ltd., Hohhot, China; ^5^Hangzhou Hailu Medical Technology Co., Ltd., Hangzhou, China; ^6^Institute of Food Science, Zhejiang Academy of Agricultural Sciences, Hangzhou, China

**Keywords:** *Bifidobacterium lactis* BL-99, fructooligosaccharide, constipation, intestinal microbiota, *in vitro* fermentation

## Abstract

Probiotics and prebiotics relieve constipation by altering the composition of the intestinal microbiota. However, their synergistic mechanism of action remains unclear. Herein, an *in vitro* fermentation model was constructed to examine the synergistic effects of *Bifidobacterium lactis* BL-99 and fructooligosaccharide (FOS) on the regulation of intestinal microbiota from a population with constipation. The utilization of FOS was promoted by BL-99, and the increase rate being 22.33%. Relative to the BL-99 and the FOS groups, the BL-99_FOS group showed a highly significant increase in acetic acid content (*P* < 0.01) and a marked decrease in CO_2_ and H_2_S contents (*P* < 0.01) in the fermentation broth. In addition, the BL-99_FOS combination significantly changed the structure of the intestinal microbiota, enhanced the relative abundances of beneficial bacteria that relieved constipation, including *Bifidobacterium, Fecalibacterium, Lactobacillus, Subdoligranulum*, and *Blautia*, and decreased those of the harmful bacteria, including *Bilophila* and *Escherichia-Shigella*. These findings suggested that BL-99 and FOS synergistically regulated the composition and structure of the intestinal microbiota from the population with constipation and increased acetic acid and decreased CO_2_ and H_2_S levels, thereby providing a theoretical basis for the application of synbiotics.

## Introduction

Constipation is a common intestinal disorder (1) afflicting 10 to 20% of the global population (2). It threatens intestinal health by reducing bowel movements and prolonging the duration of defecation, leading to the proliferation of harmful bacteria in the colon (3). In addition, constipation increases the risk of cardiovascular diseases, including atherosclerosis and cardiac arrhythmias, thereby adversely affecting the daily life of these patients (4). At present, the commonly prescribed drugs currently to treat constipation are laxatives, in particular, the osmotic or secretory laxatives. However, these exert various side effects, including dizziness, dehydration, and constriction of the coronary arteries (5). Consequently, more than 50% of patients are not fully satisfied with this therapy (6).

In recent years, probiotics, prebiotics, and synbiotics have been used increasingly in clinical practice to improve constipation in humans. They are economical, safe, and bear no side effects on the human body. Probiotics are live microorganisms that provide health benefits to the host, including *Lactobacilli* and *Bifidobacteria* (7). Previous studies confirm that *Bifidobacterium bifidum* GCL2505, *Bifidobacterium bifidum* BGN4, and *Bifidobacterium longum* BORI (8) settle in the intestine, thus regulating the intestinal microbiota of the host, wherein they are utilized by the microorganisms to produce acid, thus lowering the intestinal pH value and promoting the health of the host (9). Prebiotics are substrates that provide health benefits to the host and are selectively utilized by host enteric microorganisms (10). Fructooligosaccharide (FOS), a high-quality dietary fiber, is a bifidogenic factor that significantly promotes the growth and multiplication of *Bifidobacterium* (11). Synbiotics are a mixture of prebiotics and probiotics. Previous reports suggest that specific combinations of synbiotics can regulate the balance of the body’s intestinal microbiota, inhibit the colonization and multiplication of pathogenic bacteria, and promote gastrointestinal motility, thus contributing to relieving constipation (12, 13). Waitzberg et al. have confirmed that the combination of *Bifidobacterium lactis* HN019 and FOS improves the voiding parameters and the intensity of constipation in women with chronic constipation (12). However, The mechanism of synbiotics in improving constipation is not clear.

*Bifidobacterium animalis* subsp. *lactis.* BL-99 (CGMCC No. 15650), a non-pathogenic strain, was isolated from the feces of a two-year-old Chinese healthy infant and is known to reduce intestinal inflammation (14). Herein, we examined the effects of *Bifidobacterium lactis* BL-99 and FOS on the regulation of intestinal microbiota from a population with constipation using an *in vitro* fermentation model. These findings are expected to provide a theoretical basis for the development of BL-99 and FOS synbiotic products.

## Materials and Methods

### Sample Collection and Participants

In this study, a total of 10 individuals with constipation were included. The inclusion criteria were as follows: those who met the Rome IV diagnostic criteria for constipation (15); age 20–50 years; Wexner scale requirements (score out of 30, with a positive correlation between score and severity of constipation (16), and defecating frequency of 1–3 times/week. Following were the exclusion criteria: use of any probiotic product in 2 weeks prior to the beginning of the study; suffering from gastrointestinal, neurological, cardiovascular, endocrine, renal, and/or other chronic diseases that may affect intestinal motility; suffering from chronic diarrhea; those who underwent gastric banding for weight loss or gastric bypass surgery and use of conventional laxatives, and pregnant or lactating women. The study design received the approval of the Ethics Committee of Hangzhou Normal University (approval number: 20190061). All participants in this study participated voluntarily and provided signed informed consent.

### Stool Sample Collection and *in vitro* Fermentation Assay

The fecal samples from constipated person were collected in sterile stool collection tubes, transported to the laboratory at low temperature, and stored at −80°C until subsequent use. All samples were divided into four groups for the *in vitro* fecal fermentation assay as described previously by Ignacio et al. with appropriate modifications (17). Specifically, 0.8 ± 0.02 g of the sample was placed in a HALO-F100 fecal processor (Suzhou Hailu Biotech, Jiangsu, China) to obtain a 10% fecal suspension. Subsequently, 0.5 mL of the clarified suspension was injected into the fermentation flasks containing YCFA medium (18). Samples were divided into the following 4 groups: blank control (no additional addition), BL-99 (5 × 10^9^ cfu/mL), FOS (concentration of 4 g/L, purchased from Baolingbao Biology, Shandong, China), and BL-99_FOS (BL-99 concentration of 5 × 10^9^ cfu/mL and FOS concentration of 4 g/L). All samples were tested after 24 h of fermentation at 37°C.

### Determination of Fructooligosaccharide Degradation Rate

The FOS degradation rate was determined using thin-layer chromatography following the method described previously by Wang et al. with appropriate modifications (18). Briefly, 0.2 μL of the sample was placed at the marked position on the chromatography plate. Subsequently, the blow-dried chromatography plate was quickly immersed in the middle of the chromatography tank, covered with a lid, and left until the level of the developing solvent reached the top of the plate; next, it was removed and quickly blown dry. Further, the dried plate was quickly submerged in the color development agent. Next, the liquid on the plate was blown dry to ensure color development. The scanned images were processed using the scanner through the “TLC” software. Finally, the FOS degradation rate was calculated using the following equation.


FOSdegradationrate(%)=

$$\frac{0\,h\,\mathrm{avarage}\,\mathrm{gay}\,\text{value}-24\,h\,\mathrm{avarage}\,\mathrm{gay}\,\mathrm{value}}{0\,h\,\mathrm{avarage}\,\mathrm{gay}\,\mathrm{value}}\times 100\%$$

### Gas Detection

The H_2_, CO_2_, CH_4_, and H_2_S concentrations and total gaseous release from *in vitro* fermentation were measured using a gas analyzer (HL-QT01, Hailu Biotech, Hunan, China) following the procedure described by Wang et al., with appropriate modifications (19). Specifically, the gas analyzer was calibrated by detecting the gasses in a control medium without any inoculum. The fermented gas was made to enter the gas analyzer through a sampling tube, wherein the gas content was determined by a gas sensor. Finally, the measurements were analyzed using the “Multi-Gas Analyzer.exe software.

### Detection of Short-Chain Fatty Acids

The short-chain fatty acid (SCFAs) levels in various groups of fermentation broth were determined by gas-phase method according to the protocol described by Henrique-Bana et al. with appropriate modifications (20). Briefly, 0.5 mL of 10% fecal suspension was mixed with 0.1 mL of 25% metaphosphoric acid for 24 h at −20°C. Subsequently, this mixture was thawed, centrifuged for 20 min at 4°C, 3000 *0067*, and filtered using a 0.22 μm membrane for further use. A gas chromatograph (GC, Shimadzu, GC-2010 Plus, Kyoto, Japan) equipped with the DB-FFAP column (30m × 250 μm × 0.25 μm, Agilent Technologies, Inc.) was used for measuring SCFA content. Measurements included levels of total acid, propionic acid, acetic acid, and butyric acid (purity [standards of propionic acid, acetic acid, and butyric acid] >99%, chromatographic grade, purchased from Dr. Ehrensorfer Standards, Germany), with 2-ethylbutyric acid as the internal standard.

The gas chromatography conditions were as follows: Agilent DB-FFAP capillary column (30 m × 0.25 mm × 0.25 μm, column temperature of 75°C, Agilent Technologies); 20°C/min to 180°C for 1 min and 50°C/min to 220°C for 1 min; injection port temperature: 250°C; injection volume; 1.0 μL; split ratio: 5:1; carrier gas type: high purity nitrogen; detector type: FID; temperature: 250 °C; make-up gas concentration: 20 mL/min; air flow rate: 300 mL/min, and hydrogen flow rate: 30 mL/min.

### 16S rRNA Gene Amplification and Analyses of MiSeq Sequencing

Using the MOBIO Power Fecal DNA Isolation Kit (Qiagen, Germany), the total fecal DNA was extracted following the protocol described by Marsaux B and Nicolucci A C (21, 22), with appropriate modifications. In the V3-V4 region of the 16S rRNA gene, the sample-specific barcode sequences were amplified using the primer pair, 806R (5′-GTGGACTACHVGGGTWTCTAAT-3′) and 338F (5′-GTACTCCTACGGGAGGCAGCA-3′). The resulting amplicon libraries (Roche KAPA Library Quantification Kit) were equally combined and subjected to quantification and further sequenced on the Illumina MiSeq platform in the PE300 mode. Using the Quantitative Insights into Microbial Ecology version 2.0 (QIIME2) pipeline, we identified the sequences by barcoding. Sequences of acceptable quality (i.e., minimum mass fraction = 25, min/max length = 200/750, no mismatches in primer sequences, and no base ambiguity) were retained. Further, these were clustered into operational taxonomic units (OTUs) by a redundancy algorithm embedded in the DADA2 package and classified using the Silva database (version: SSU138.1). Subsequently, we constructed phylogenetic trees for the representative sequences and calculated the β-diversity by weighted principal coordinate analysis (PCoA), and α-diversity using the Shannon index. Species correlations with SCFAs and gasses were shown using correlation heatmap plots based on the Spearman rank correlation coefficients.

### Statistical Analysis

Data were expressed as mean ± standard deviation (M ± SD). All statistical analyses were performed on the SPSS 23.0 software (SPSS Inc. Chicago, IL, United States). The one-way analysis of variance was used to make comparisons between samples. *P* < 0.05 was the indicator of statistical significance. The graphs were plotted using (GraphPad Software Inc., San Diego, CA, United States) GraphPad Prism 8.

## Results

### Basic Information of Participants

The ten adult female participants in the study had an average age of 28.9 ± 5.2 years, mean height of 162.8 ± 7.1 cm, and mean weight of 59 ± 10.6 kg. They complied with the Wexner scale (out of 30) after inclusion. The basic information of the participants is listed in Table 1.

**TABLE 1:** Basic information of participants.

Participant No.	Age (years)	Height (cm)	Body weight (kg)	Score
1	33	162	68	14
2	25	156	55	12
3	33	164	62	11
4	32	155	58	12
5	29	163	49	17
6	34	160	55	10
7	35	158	52	14
8	25	179	85	9
9	21	161	53	15
10	22	170	53	9

### Fructooligosaccharide Degradation Rate

**FIGURE 1:**
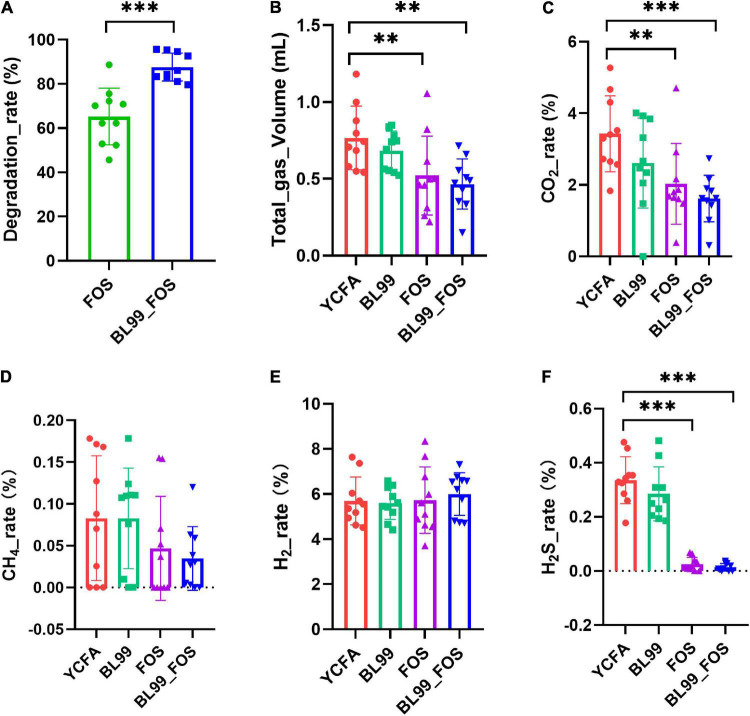
FOS degradation rate **(A)**, total gas production **(B)**, and gas production ratio (CO_2_ **(C)**, CH_4_ **(D)**, H_2_ **(E)**, and H_2_S **(F)** in various groups. Statistical significance was calculated by one-way ANOVA with LSD test, * was used to mark the significance between different groups, and no mark means there is no significance.

The FOS degradation rate in the fermentation broth after *in vitro* fermentation is shown in Figure 1A. After 24 h of fermentation, the FOS degradation rates in FOS and BL-99_FOS groups were 65.25 ± 12.82% and 87.58 ± 6.30%, respectively. These markedly significant differences (*P* < 0.001) in the FOS degradation rates between the two groups indicated that BL-99 could utilize FOS.

### Total Gas Production and the Percentages of CO_2_, CH_4_, H_2_, and H_2_S

Gas production was measured in each group after 24 h of *in vitro* fermentation (Figures 1B–F). Relative to the YCFA group, the CO_2_ production in the FOS group was significantly lower (*P* < 0.01), whereas that in the BL-99_FOS group was highly significantly lower (*P* < 0.001). CO_2_ production was also lower in the BL-99 group, however, this difference was not statistically significant. CH_4_ content decreased in the BL-99, FOS, and BL-99_FOS groups, however, the comparisons were not significant. H_2_ content increased in the BL-99, FOS, and BL-99_FOS groups but the differences were not statistically significant. H_2_S production was lowered by a highly significant level in the FOS and BL-99_FOS groups (*P* < 0.001). H_2_S production was reduced in the BL-99 group, however, the difference was not statistically significant. The total production of the four gasses was highly significantly lowered in the FOS and BL-99_FOS groups (*P* < 0.01).

### Short-Chain Fatty Acids Content

**FIGURE 2:**
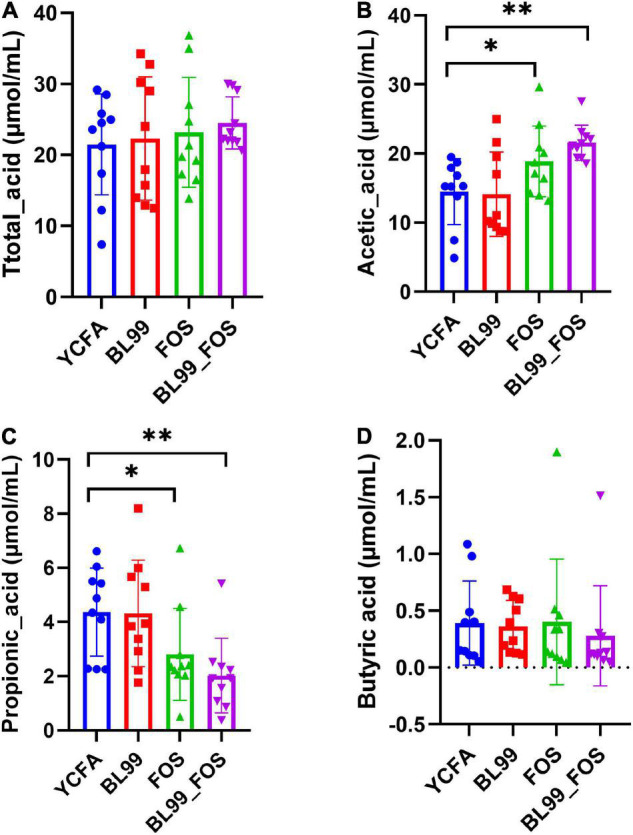
Total acid **(A)**, acetic acid **(B)**, propionic acid **(C)**, and butyric acid **(D)** contents in different groups. Statistical significance was calculated by one-way ANOVA with LSD test, *P < 0.05, **P < 0.01, and ***P < 0.001 were considered with significant difference.

After completion of the *in vitro* fermentation, the contents of SCFAs, including total acid(A), acetic acid(B), propionic acid(C), and butyric acid(D) were measured in the fermentation broth (Figures 2A–D). Relative to the YCFA group, the acetic acid content was markedly high in the FOS group (*P* < 0.05) and even higher in the BL-99_FOS group (*P* < 0.01). The propionic acid content reduced significantly in the FOS group (*P* < 0.05) and decreased further significantly in the BL-99_FOS group (*P* < 0.01). There were no significant differences in the contents of butyric acid and total acids among the BL-99, FOS, and BL-99_FOS groups (*P* > 0.05). However, the YCFA group showed the lowest total acid content (21.48 ± 7.11 μmol/mL), while the BL-99_FOS group exhibited the highest value of the total acid content (24.52 ± 3.66 μmol/mL).

### Effects of BL-99 and Fructooligosaccharide on Intestinal Microbiota in Patients With Constipation

16S rRNA high-throughput sequencing was performed for 40 samples from different groups on the Illumina Miseq double-end sequencing platform. A total of 3,315,045 raw sequences were obtained after filtering and splicing. The average number of reads per sample was 82,876 ± 275 reads. Subsequently, all chimeric sequences were excluded using the Usearch tool by the Uchime algorithm. The remaining sequences were clustered into OTUs at 97% similarity cut-off and unique representative OTU sequences were identified. After sequence annotation, OTUs with no results and those annotated as archaea were removed. The remaining OTUs were used for further analyses. Finally, in all samples, a total of 608 OTUs categorized into 9 phyla, 18 classes, 39 orders, 66 families, 146 genera, and 280 species were annotated.

### Analysis of Alpha-Diversity

**FIGURE 3:**
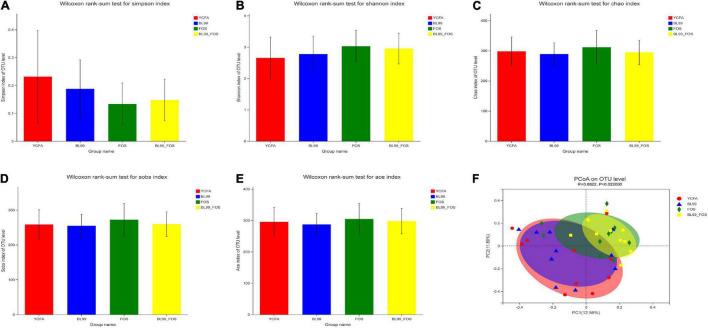
PCoA plots for α-diversity indices in different groups, including the Simpson index **(A)**, Shannon index **(B)**, Chao index **(C)**, Sobs index **(D)**, and Ace index **(E)**, and β-diversity analysis **(F)**.

The alpha diversity in the gut microbial composition of the different groups was evaluated using the OTUs. As shown in Figures 3A–E, no statistically significant differences in the α-diversity indexes among the groups (*P* > 0.05) were found. As compared with to the YCFA group (0.23 ± 0.12), the Simpson index was reduced in the BL-99 group (0.19 ± 0.13), the FOS group (0.13 ± 0.09), and the BL-99_FOS group (0.15 ± 0.12), while the Ace, Shannon, Chao, and Sobs indices were all higher in FOS and BL-99_FOS groups. These results suggested that FOS and BL-99_FOS could significantly increase the microbial alpha diversity of the intestinal microbiota.

### Analysis of Beta-Diversity

Principal coordinate analysis based on Bray–Curtis distances was performed to identify the beta-diversity in the intestinal microbiota at the OTU level to further examine the differences in the overall microbial composition among the four groups (Figure 3F). The *P*-value for PCoA cluster analysis was 0.022 (*P* < 0.05), indicating that there were statistically significant differences in the overall microbial composition among groups. As shown in Figure 3F, a large degree of dispersion within the YCFA group, and varying degrees of aggregation in BL-99, FOS, and BL-99_FOS groups were inferred. In addition, the microbial compositions in FOS and BL-99_FOS groups were significantly different from those in the YCFA group, suggesting that constipation led to changes in the structure of the intestinal microbiota. However, FOS and BL-99_FOS could induce new homeostasis in the previously disturbed intestinal microbiota.

### Species Composition

**FIGURE 4:**
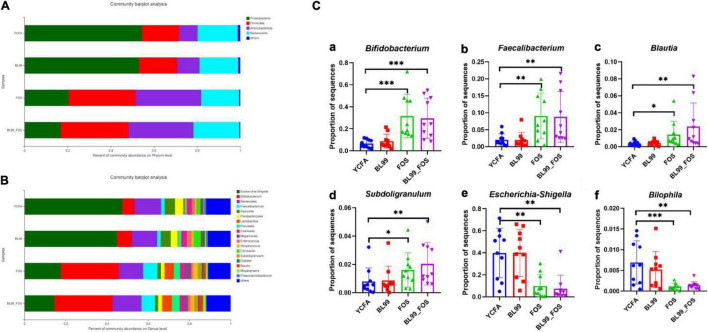
Histogram of species composition at the phylum **(A)** and genus **(B)** level and relative abundance of dominant species in different groups **(C)** [Bifidobacterium **(a)**; Faecalibacterium **(b)**; Blautia **(c)**; Subdoligranulum **(d)**; Escherichia-Shigella **(e)**; and Bilophila **(f)**]. Statistical significance was calculated by one-way ANOVA with LSD test, *P < 0.05, **P < 0.01, and ***P < 0.001 were considered with significant difference.

The differences in intestinal microbiota compositions among YCFA, BL-99, FOS, and BL-99_FOS groups were compared at the genus and phylum levels using OTU taxonomy and composition analysis (Figure 4). At the phylum level (Figure 4A), the intestinal microbiota of the population with constipation mainly comprised of *Firmicutes*, *Bacteroidetes*, *Proteobacteria*, and *Actinobacteria*. Relative to the YCFA group, the relative abundances of *Firmicutes* and *Actinobacteria* were enhanced, while that of *Proteobacteria* reduced in the BL-99, FOS, and BL-99_FOS groups. As compared to the BL-99 group, the relative abundances of *Firmicutes* and *Actinobacteria* were increased, while that of *Proteobacteria* reduced in the FOS group. Additionally, these effects were more pronounced in the BL-99_FOS group.

Relative abundances of the intestinal microbiota at the genus level were observed (Figure 4B). We focused on the microorganisms with relative abundances greater than 1%, which included *Escherichia-Shigella*, *Bifidobacterium*, *Bacteroides*, *fecalibacterium*, *Klebsiella*, *ParaBacteroides*, *Lactobacillus Prevotella*, *Collinsella*, *Megamonas*, *Enterococcus*, *Streptococcus*, *Citrobacter*, *Subdoligranulum*, *Dialister*, *Blautia*, *Megasphaera*, and *Phascolarctobacterium*. Subsequently, the relative abundances of dominant species at the genus level were compared among different groups (Figure 4C). Relative to the YCFA group, the relative abundances of *Bifidobacterium* and *fecalibacterium* showed a highly significant increase in the FOS and BL-99_FOS groups (both *P* < 0.001), whereas those of *Blautia* and *Subdoligranulum* were markedly enhanced in the FOS group (*P* < 0.05); *Blautia* and *Subdoligranulum* in the BL-99_FOS group were further significantly higher (*P* < 0.01); *Escherichia-Shigella* in FOS and BL-99_FOS groups showed a markedly significant decrease (*P* < 0.01); *Bilophila* in the FOS group increased significantly (*P* < 0.001), and *Bilophila* in the BL-99_FOS group showed a markedly significant increase (*P* < 0.01).

### Correlational Analysis

**FIGURE 5:**
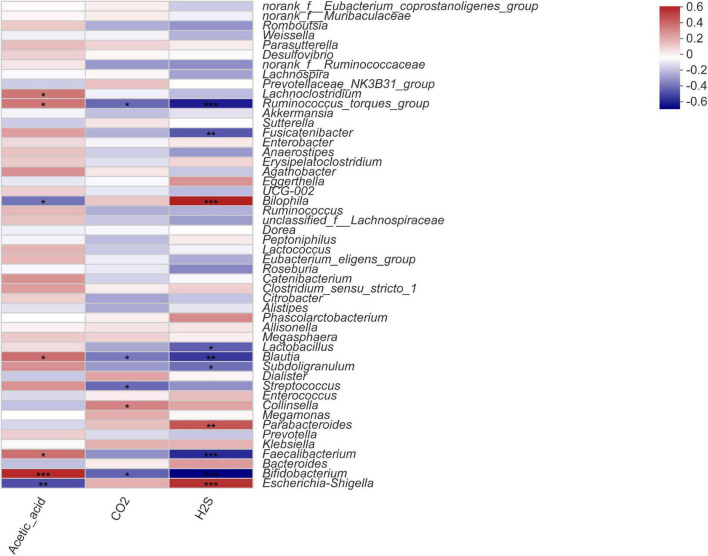
Spearman correlation heatmap for microorganisms at the genus level with acetic acid, CO_2_, and H_2_S content. Statistical significance was calculated by one-way ANOVA with LSD test, *P < 0.05, **P < 0.01, and ***P < 0.001 were considered with significant difference.

To investigate the correlation between the intestinal microbiota, gas composition, and SCFAs, the Spearman correlation coefficient was used to evaluate the association between microbial composition at the genus level and significantly variable acetic acid, CO_2_, and H_2_S levels (Figure 5). The Spearman correlation heatmap showed that *Bifidobacterium* was markedly significantly positively correlated with acetic acid content (*P* < 0.001) and highly significantly negatively correlated with H_2_S content (*P* < 0.001). *Fecalibacterium* was significantly positively correlated with acetic acid content (*P* < 0.05) and highly significantly negatively associated with H_2_S content (*P* < 0.01); *Blautia* was significantly positively related to acetic acid content (*P* < 0.05) and highly negatively correlated with CO_2_ and H_2_S contents (*P* < 0.01). *Escherichia-Shigella* was highly significantly negatively correlated with acetic acid content (*P* < 0.01) and highly positively correlated with H_2_S content (*P* < 0.001). *Bilophila* was highly significantly negatively correlated with acetic acid content (*P* < 0.05) and highly positively correlated with H_2_S content (*P* < 0.001), and *Lactobacillus* was significantly negatively correlated with H_2_S content (*P* < 0.001).

## Discussion

Probiotic bacteria such as *Bifidobacterium*, *Enterococcus*, and *Lactobacillus* can utilize FOS. In general, FOS is mainly catabolized in two ways: FOS can be hydrolyzed by extracellular fructosidase, and the resulting products of hydrolysis, fructose and glucose, are transported into the cell *via* the phosphoenolpyruvate-sugar phosphotransferase (PTS)system for production of fructose-6-phosphate and glucose-6-phosphate, respectively for glycolysis, such as *Lactobacillus pentosus* (23); FOS is transported intact into the cell *via* the PTS transport proteins, it is hydrolyzed by intracellular fructosidases; subsequently, the monosaccharides obtained after hydrolysis are subjected to the glycolytic processes, for example *Lactobacillus* acidophilus, *Lactobacillus plantarum*, *Lactobacillus salivarius*, and most *bifidobacteria* (24, 25). However, not all probiotics can utilize FOS. For instance, *Lactobacillus acidophilus*, *Lactobacillus plantarum*, and *Lactobacillus casei* can use FOS, while the widely studied probiotic strain, LGG, is unable to utilize FOS. Moreover, *Lactobacillus bulgaricus*, commonly used for yogurt production, cannot utilize FOS (26). The results of this study suggested that BL-99_FOS degraded FOS at a highly significant enhanced rate relative to FOS (*P* < 0.001), thus indicating that BL-99 could utilize FOS and the combination, BL-99_FOS, needs further research potential.

When food enters the gastrointestinal tract, most of the nutrients are digested and absorbed. Some unabsorbed carbohydrates, such as oligosaccharides, cellulose, and starch, enter the colon and are decomposed by the intestinal microbiota, herein, to produce various gaseous components such as H_2_, CH_4_, CO_2_, and H_2_S (27). These gasses are mainly produced by anaerobic microbiota through partial or complete fermentation of carbohydrates (28). Therefore, in this study, the contents of these gasses in fermentation broth were measured *in vitro*. Furthermore, relevant previous studies show that gasses produced by microbiota are closely associated with clinical functional gastrointestinal disorders. They not only affect the sensory-motor functions of the intestine (29), but also stimulate intestinal peristalsis and shorten colonic transport time either directly or indirectly by lowering the intestinal pH (30). However, excessive gaseous production in the intestine can also cause abdominal distension (31). Herein, we found that the BL-99_FOS synbiotic group could significantly reduce CO_2_ content (*P* < 0.01) and total gas production (*P* < 0.01), thereby indicating that BL-99_FOS synbiotics could regulate gas composition through the intestinal microbiota.

Short-chain fatty acids are the end products of carbohydrate metabolism of intestinal microorganisms. Several reports confirm that SCFAs have crucial regulatory functions for physiological activities and maintenance of the intestinal microenvironment in hosts. Butyric acid, propionic acid, and acetic acid account for more than 95% of the total SCFA content in the colonic segment (32). Therefore, this study focused on investigating the effects of FOS and BL-99 on the content of these three acids. Acetic acid has multiple physiological functions. Yin et al. (33) report that acetic acid can lower intestinal pH value and act as an antibacterial agent. In addition, it can serve as a source of energy for the peripheral blood cells of the intestine and the liver, and as a signaling molecule in the metabolic pathways, including gluconeogenesis and lipogenesis for monitoring the functions of the colon (34). The results of the present study indicated that BL-99 and FOS significantly increased the acetic acid content in the fermentation broth. These findings suggested that BL-99_FOS synbiotics could promote the production of acetic acid, thus benefiting organismal health.

The proportion of beneficial bacteria in the gut is positively correlated with the health status of an individual and only 15% beneficial bacteria (e.g., *Lactobacillus* and *Bifidobacterium*) are found in the gut of patients with constipation (35). Constipation causes an increase in the abundances of pathogenic bacteria, such as *Enterobacter* and *Enterococcus*. Herein, we also found that the relative abundances of beneficial bacteria such as *Bifidobacterium*, *fecalibacterium*, *Blautia*, and *Subdoligranulum* were significantly lower, while those of harmful bacteria, such as *Escherichia-Shigella* and *Bilophila* was significantly higher in the YCFA group as compared to the other groups. The extracellular polysaccharide of *Bifidobacterium lactis* can inhibit the growth of *Clostridium perfringens* and resist the dysbiosis of the intestinal microbiota caused by mucosal damage in mice. Thus, *Bifidobacterium bifidum* colonized in the intestinal tract can exert a barrier effect with the host microbiota, thus contributing to intestinal health (36). *Fecalibacterium*, *Blautia*, and *Subdoligranulum*, like probiotics, can contribute to relieving constipation symptoms by increasing bacterial fermentation end-products, thus improving the intestinal lumen environment, promoting the output of SCFAs and anti-inflammatory factors, regulating intestinal gas composition, and improving the metabolism of bile acids, vitamins, and ascorbic acid (37). In addition, these can also alleviate constipation by affecting the levels of 5-HT and brain-derived neurotrophic factors (38). Human intestinal bacteria can ferment prebiotic FOS, thus benefiting organismal health (39). Probiotics can promote the utilization of FOS by intestinal microbiota (40). Parhi et al. (41) show that *Lactobacillus casei* can promote the utilization of FOS by fecal microbes, thereby altering the intestinal microbial composition. The results of the population trial showed that *Bifidobacterium longum* W11 combined with FOS synbiotics could improve the defecation frequency and reduce the colonic transport time of constipated patients (42). A lot of researcher suggested that FOS and *Bifidobacterium* were associated with relieved constipation symptoms, promoted the increase of beneficial bacteria and decrease of harmful bacteria in the intestinal tract (43, 44). The present study also demonstrated that FOS could be degraded by human intestinal microorganisms. Additionally, BL-99 could promote the utilization of FOS, thus further altering the overall structure of the intestinal microbiota.

## Conclusion

The effects of BL-99 and FOS synbiotics on the regulation of intestinal microbiota from patients with constipation were investigated using an *in vitro* fermentation model. The findings of this study showed that BL-99 promoted the utilization of FOS in the intestine and BL-99_FOS synbiotics could significantly enhance the acetic acid content in the fermentation broth while decreasing the CO_2_ and H_2_S contents. In addition, the synbiotics changed the overall structure of the intestinal microbiota, increased the abundances of acetic acid-producing beneficial bacteria, including *Bifidobacterium*, *fecalibacterium*, and *Blautia*, and decreased those of the H_2_S-producing harmful bacteria such as *Escherichia-Shigella* and *Bilophila*. Thus, these findings are expected to provide a theoretical basis for the use of synbiotics.

## Data Availability Statement

The original contributions presented in the study are included in the article/supplementary material, further inquiries can be directed to the corresponding authors.

## Ethics Statement

The animal study was reviewed and approved by the Ethics Committee of Hangzhou Normal University (approval number: 20190061).

## Author Contributions

QZ, WZ, W-LH, and RW conceived and designed the experiments. QZ, YZ, SD, TW, and XW conducted the experiments. QZ, YZ, W-HL, CZ, and SS conducted the analysis. QZ, W-LH, and RW interpreted the results and wrote the manuscript. All authors read and approved the final manuscript.

## Conflict of Interest

SD, W-HL, and W-LH were employed by the company Inner Mongolia Dairy Technology Research Institute Co., Ltd., and Inner Mongolia Yili Industrial Group Co., Ltd. TW was employed by the company Hangzhou Hailu Medical Technology Co., Ltd. The remaining authors declare that the research was conducted in the absence of any commercial or financial relationships that could be construed as a potential conflict of interest.

## Publisher’s Note

All claims expressed in this article are solely those of the authors and do not necessarily represent those of their affiliated organizations, or those of the publisher, the editors and the reviewers. Any product that may be evaluated in this article, or claim that may be made by its manufacturer, is not guaranteed or endorsed by the publisher.
